# Exergaming: Feels good despite working harder

**DOI:** 10.1371/journal.pone.0186526

**Published:** 2017-10-23

**Authors:** Kate Glen, Roger Eston, Tobias Loetscher, Gaynor Parfitt

**Affiliations:** 1 School of Psychology, University of South Australia, Adelaide, South Australia; 2 Alliance for Research in Exercise, Nutrition and Activity (ARENA), Sansom Institute for Health Research, School of Health Sciences, University of South Australia, Adelaide, South Australia; University of Rome, ITALY

## Abstract

Strategies to encourage exercise have led to research on cycle ergometer ‘exergaming’, as a means of enhancing exercise enjoyment. This research has typically prescribed the exercise intensity and used one exercise mode. The aim of this study was to compare self-selected exercise intensity on a cycle ergometer with and without exergaming modes activated. A total of 20 participants aged between 18–40 years (*M* = 24.2 ± 5.9) completed a sub-maximal exercise test. Participants returned two days later to complete one 45 minute session of self-selected exercise with 15 minutes in each of ‘control’ (standard ergometer), ‘track’, and ‘game’ modes, with order randomized. Heart rate, work rate, perceived exertion, and affective valence were recorded during exercise. Dissociation and enjoyment were recorded in the rest interval between each mode. Participants exercised above ventilatory threshold (VT) in all three modes (track, *M* = 9.5 *±* 12%; game, *M* = 6.2 *±* 12%; and control, *M* = 4.4 *±* 14% above VT) and at higher work rates (*P* < 0.05) exergaming (track, *M* = 94.5 *±* 27.9; game, M = 96.2 *±* 32.8 watts) than control (M = 86.6 *±* 26.5 watts). Despite exercising at a higher intensity, participants perceived exercise during the exergaming modes to be most pleasant (*P* < 0.01), with greater enjoyment *(P <* 0.01) and dissociation *(P <* 0.01) in the game than track mode, and both modes higher on these variables than the control mode. Findings support the use of exergaming as a strategy to encourage individuals to exercise, with participants choosing to work harder physiologically, but reporting more positive psychological responses during and following the exercise.

## Introduction

Global physical inactivity levels have been described as pandemic and have attributed to approximately 3.2 million deaths [1]. Strategies and approaches to help change this statistic are necessary. Research has demonstrated a link between the acute affective (pleasant/unpleasant) responses to exercise and future exercise participation [2, 3]. Further, in their exercise prescription guidelines, the American College of Sports Medicine [4] indicate that negative affect “can act as a deterrent to continued participation” (p374). Evidence for methods and modalities of exercise that avoid negative affect and support a positive affective response is therefore important.

Affective responses to exercise have been shown to be both intensity and prescription dependent [5–7]. As exercise intensity increases beyond the ventilatory threshold (VT: an intensity related to the imbalance of aerobic and anaerobic metabolism) affective responses decline rapidly. According to the Dual-Mode Model [8] this is due to the interplay between cognitive processes (personality, goals, exercise efficacy etc.) and physiological cues from the body during exercise. At low exercise intensity, the cognitive processes dominate, with affective responses generally positive, but as exercise intensity increases beyond the level of physiological homeostasis, the physiological cues dominate and affective responses become homogenously negative [5, 9]. Evidence from studies including children [10, 11], young adults [12], and older adults [13] support this pattern. Further, when the exercise intensity is prescribed, rather than self-selected or a preferred intensity, the affective responses have been shown to be more negative [6, 7].

High variability in affective response has been recorded close to the individual’s VT [14] when the intensity is prescribed. However, this variability in affective response is reduced when the individual is allowed to self-select the intensity [5, 15]. Individuals typically commence exercise at an intensity (assessed using heart rate and perceived exertion) below their VT, but then choose to increase it during exercise [9, 15], with the resulting intensity close to VT [5]. It has recently been demonstrated that exercise intensity which is prescribed around the VT elicited significantly (p < 0.05) greater improvements in maximal oxygen uptake (VO_2_max) and attenuated individual variation in VO_2_max training responses when compared to exercise training prescribed at a percentage of maximal heart rate reserve [16].

When self-selecting the intensity, the individual is thought to use the interplay between cognitive and physiological cues, and select an intensity that accommodates his/her personality, goals, efficacy, and interpretation of cues [5]. Findings from self-selected exercise studies consistently show that affect remains positive and stable during both aerobic exercise [15, 17, 18] and resistance training [6, 19]. Additionally, the process of self-selection of exercise intensity may foster a sense of perceived autonomy, which according to Self-Determination Theory, is one of the basic psychological needs required to develop intrinsic motivation [20]. Supporting an individual to use self-selection of intensity may therefore not only result in positive affect, but be important motivationally to maintain regular engagement in exercise [3].

Along with self-selection, the use of distraction can aid in the maintenance of the positive affective response. Distracting techniques previously identified include listening to music [21] or music and video [22]. Attentional dissociation has been identified as an effective technique for distracting individuals from noxious physiological cues and maintaining positive affect [23] by reducing awareness and diverting attention away from the cues [24]. Studies measuring affect provide evidence for a decline in pleasure when reaching VT where the attention shifts towards the physiological cues and away from dissociation [25].

Attentional models, for example Tenenbaum’s [26] Social-Cognitive Model of Attention, and Leventhal & Everhart’s [27] Parallel Processing Model, indicate that the ability to dissociate is exercise intensity dependent, with both proposing that the ability to divert attention is inhibited beyond a critical level of exertion. Research by Tenenbaum and colleagues [28, 29] supports this with attention becoming more internally focused as the exercise intensity increases, although research demonstrates that music and music-video conditions were still effective distractors at 10% above VT [23].

The provision of entertainment through technology (particularly television, video games, and exergaming) provides a means of distracting the individual while exercising. Exergaming (the integration of exercise with video gaming) has gained popularity as a strategic approach that may encourage otherwise sedentary and obese children and adolescents to exercise [30], with increases in energy expenditure reported when exergaming, compared to non-active video gaming [31]. Cycle ergometers, interfaced with video (e.g. cycle tracks) and gaming technology are prevalent in fitness centres and gyms, with data indicating increased adherence to exercise on such ergometers and improvements to health, compared with standard ergometers in young men [32]. A recent study provides further support, with higher energy expenditure and enjoyment in young adults when using exergaming rather than standard ergometers [33]. However, in the study by Monedero and colleagues [33], exercise intensity was prescribed to 55% of each participant’s peak power output, and while enjoyment was assessed, there was limited assessment of other relevant psychological factors (e.g. distraction and affective valence). In addition, only one game mode was considered. There are no data objectively evaluating what individuals choose to do (the intensity they select) on such cycle ergometers and the corresponding acute physiological and psychological effects of exergaming using different game modes. With game modes differing in the amount of immersion (interaction and decision making), a comparison of modes is of interest, with the expectation that there will be greater distraction and enjoyment the more immersive the game. For example, in some modes participants can follow tracks and trails with distracting scenes and cyclists. In others, they can interact more directly, change direction, and chase objects for points or medals. So, although studies have compared prescribed intensity exercise on standard and exergaming ergometers [33–35], research has not systematically examined what intensity individuals choose when allowed to self-select the intensity (which would be more ecologically valid), and measured both the physiological and psychological (affective, dissociative and enjoyment) effects associated with the different modes.

The objective of this study was to examine the effect of self-selected exercise in *control* mode (no visual distraction–a blank screen) and during exergaming with either a *track* mode (following a woodland trail) or a *game* mode (chasing dragons to accrue points). It was hypothesised that individuals would work physiologically harder during the exergaming modes, when they were distracted from the physiological cues, but maintain a positive affective response. It was also hypothesised that individuals would dissociate more and find the exergaming modes more enjoyable than the control mode, with greater enjoyment and dissociation in the game mode.

## Materials and methods

### Participants

Twenty participants (16 female), between 18–40 years (*M* = 24.15, *±* 5.9 years) were recruited to the study through posters and flyers displayed at a city university campus. Given the within subject design and that there is no evidence for males and females to respond differently to the self-selected exercise conditions, we recruited the first 20 participants who volunteered to the study. All participants provided written informed consent prior to commencing the study. Participants had no prior experience exercising on exergaming ergometers, with the majority below average fitness for their age group [4]. The study was approved by The University of South Australia Human Research Ethics Committee (Protocol number: 000003424).

### Measures

#### Work rate

During exercise, power output (watts) was recorded from the Expresso HD Recumbent bike (model 919Sw-1, Interactive Fitness, Sunnyvale, CA) and averaged for each 3-minute period of exercise.

#### Heart rate

Heart rate (HR) was measured throughout exercise (S610i, Polar Electro Oy, Kempele, Finland) and converted to %HR relative to HR at VT for analysis.

#### Rating of perceived exertion

The Borg 6–20 Rating of Perceived Exertion Scale (RPE) [36] was used to assess physical exertion, which ranges from ‘no exertion at all’ (6) to ‘maximal exertion’ (20). Participants received standardised instructions and were encouraged to focus upon their overall ‘whole body’ perceptions of exertion [36].

#### Affect

The Feeling Scale (FS) [37], a bipolar 11-point scale which ranges from -5 (very bad) to +5 (very good), was used to measure affective valence. It has been shown to correlate with other valence scales and to be sensitive to change [5].

#### Dissociation

The Single-Item Attention Scale [38] was used to assess dissociation. It is a bipolar scale that is verbally anchored to internal focus (bodily sensations), and external focus (external environment), and has been used in previous exercise studies assessing dissociation [e.g. 22].

#### Enjoyment

This was assessed with the Physical Activity Enjoyment Questionnaire (PACES), which is an 18-item, 7 point scale developed by Kendziersky & DeCarlo [39] to assess the extent of an individual’s enjoyment of physical activity regardless of exercise type. Participants were asked to rate how they felt about the activity they had just been doing. Scores ranged from 18–126, with a higher score reflective of greater enjoyment. The scale has sound test-retest reliability (0.76) and good internal consistency (0.89) [40].

#### Procedure

Participants attended the laboratory on two occasions. The first session was to familiarise the participants with the various scales, to measure height and mass, and to assess maximal oxygen uptake and the ventilatory threshold. The second session was to conduct 3 x 15-min distinct exercise conditions on the Exergaming ergometer, which formed the experimental basis for the study.

#### Session 1

Participants completed the Exercise and Sport Science Australia [41] screening tool followed by measurement of height and body mass. The HR monitor was connected via telemetry to the breath-by-breath automatic gas analysis system (Cortex Metalyzer 3B, Biophysik, Leipzig, Germany). Participants were then seated on the stationary lode cycle ergometer (Lode-Corival, Groningen, Netherlands) and sat quietly whilst the pre-exercise measures of HR were taken. The participants were familiarised with the RPE and FS scales which were displayed on the wall in front of the ergometer. Following this, a facemask (7450 Series V2, Hans Rudolph Inc, Kansas City, USA) was fitted to collect expired air.

Participants then performed a submaximal, perceptually-regulated exercise test (PRET; Eston et al [42]) which uses Borg's [36] 6–20 RPE scale and consists of 4 x 3 minute stages. The first stage involved participants cycling at RPE 9 (very light) for 3 minutes. At the completion of this stage, participants were instructed to adjust the intensity to an RPE 11 (light) on the RPE scale. The protocol was then repeated for RPE levels 13 (somewhat hard) and 15 (hard (heavy)). This sub-maximal approach was selected in line with recent developments that have demonstrated that the tests are reliable and valid and importantly avoid the negative affective response of prescribed tests [43–45]. Throughout the PRET, pulmonary ventilation (VE), gas exchange variables (oxygen uptake (VO_2_), carbon dioxide production (VCO_2_), ventilatory equivalent for oxygen (VE/VO_2_) and VCO_2_) were measured continuously to allow estimation of the ventilatory threshold, according to the method of Gaskill et al [46]. HR was also recorded continuously and extrapolated to estimate the HR at VT in order to standardise exercise intensity during the experimental conditions to an intensity which was relative to each individual’s HR at VT. The FS was also recorded in the last 15 seconds of each minute. Following the exercise test, participants were then shown how to operate the exergaming ergometer.

#### Session 2

Prior to Session 2, participants were assigned a randomly allocated order for the exercise modes to control for an order effect. Participants completed 15 minutes of exercise in each of the three modes (control mode, with blank screen; track mode [Redwood Dash 1.5 mile loop], where participants could steer [track fixed] through a woodland; and game mode [chasing dragons] where participants could change direction to catch dragons and earn points) on the Expresso HD Recumbent bike. Exercise bouts of 15 minutes were selected based on previous findings suggesting that as little as 7–10 minutes of exercise can result in significant changes in affect [47]. Furthermore, multiple bouts of physical activity of 10 minutes or more are recommended for health benefits to achieve the international guidelines of 30 min moderate activity [4, 48]. Participants were told that they would be exercising for three sets of 15 minutes and instructed that they could choose to exercise at whatever intensity they preferred and change the intensity whenever they wanted. There was no physiological or work rate data visible. Prior to commencing exercise, participants sat quietly for 5 minutes whilst a pre-exercise measure of HR was taken. HR, FS, and RPE were recorded every three minutes throughout the exercise modes. At the end of each mode, participants had a 5-minute break during which participants completed the dissociation and enjoyment questionnaires (2 min max) and heart rate recovered, before commencing the next mode.

### Data analysis

Analyses were a series of mode (control, track, game) by time (3, 6, 9, 12, 15 minute) repeated analyses of variance (ANOVA) for work rate, HR, FS and RPE. The dissociation and enjoyment data were analysed with one factor (mode) repeated measures ANOVA. All data were analysed using IBM SPSS 21.0 for Windows (SPSS Inc., Chicago, IL). Due to technical failure, HR data were lost for two participants. All other data were complete. If the assumption of sphericity was violated, degrees of freedom were corrected with the Greenhouse-Geisser epsilon. Any significant main effects or interactions were followed up by conducting repeated measures contrasts. Effect sizes were expressed as eta squared (η^2^) and were calculated from the SPSS partial η^2^ output as per the equations presented in Levine & Hullett [49], with .02, .13 and .26 interpreted as small, medium and large effects, respectively. All results are reported as means (*M*), and standard deviations (*±*). For all analyses, the level of significance was set at *p* < 0.05.

## Results

The descriptive and anthropometric measures of the participants are shown in Table 1.

**Table 1:** Means and standard deviations of participant characteristics.

	Female *n* = 16		Male *n* = 4		Total *n* = 20	
	*M*	*SD*	*M*	*SD*	*M*	*SD*
**Age (y)**	22.5	2.5	30.8	10.8	24.2	5.9
**Height (m)**	164.8	8.1	178.0	5.7	167.4	9.3
**Mass (kg)**	68.9	14.2	86.1	11.21	72.4	15.1
**Body Mass Index (kg·m**^**-2**^**)**	24.6	4.5	26.5	3.3	25.0	4.3
**Resting HR (bpm)**	92.5	13.8	79.7	2.2	89.9	13.3
**Mean HR at VT (bpm)**	135.3	10.2	122.2	16.2	132.4	12.5
**FS at VT**	1.6	1.8	2.3	1.0	1.8	1.7
**Estimated VO**_**2max**_**(ml·kg**^**-1**^ **min**^**-1**^**)**	36.9	9.2	33.9	4.8	36.3	8.5

*Note*. *n* = sample size

**Work rate.** There was a main effect for mode (*F* (2, 38) = 3.16, *P* < 0.05, η^2^ = .14) with work rate significantly higher in the game and track modes than the control mode (Table 2). The time main effect and interaction were not statistically significant.

**Table 2:** Means and SD for mode main effects.

Dependent variable	Control Mode	Track Mode	Game Mode
**Work Rate (watts)** *	86.6 ± 26.5 ^a^	94.5 ± 27.9	96.2 ± 32.8
**%HR (relative to HR at VT)**	4.4 ± 14	9.5 ± 12	6.2 ± 12
**RPE** *	12.4 ± 1.9 ^a^	13.1 ± 1.4	13.2 ± 1.2
**Affect** **	1.8 0.6 ^a^	2.1 0.6 ^b^	2.50 0.6
**Enjoyment** **	73.5 ± 21.6 ^a^	92.2 ± 18.9 ^b^	104.3 ± 9.0
**Dissociation** **	39.5 ± 27.3 ^a^	59.10 ± 27.1 ^b^	74.9 ± 22.9

Note.

** mode main effect *p* < 0.01

* mode main effect *p* < 0.05

^a^ = significantly different than track and game modes

^b^ = track significantly different to game mode

**Fig 1:**
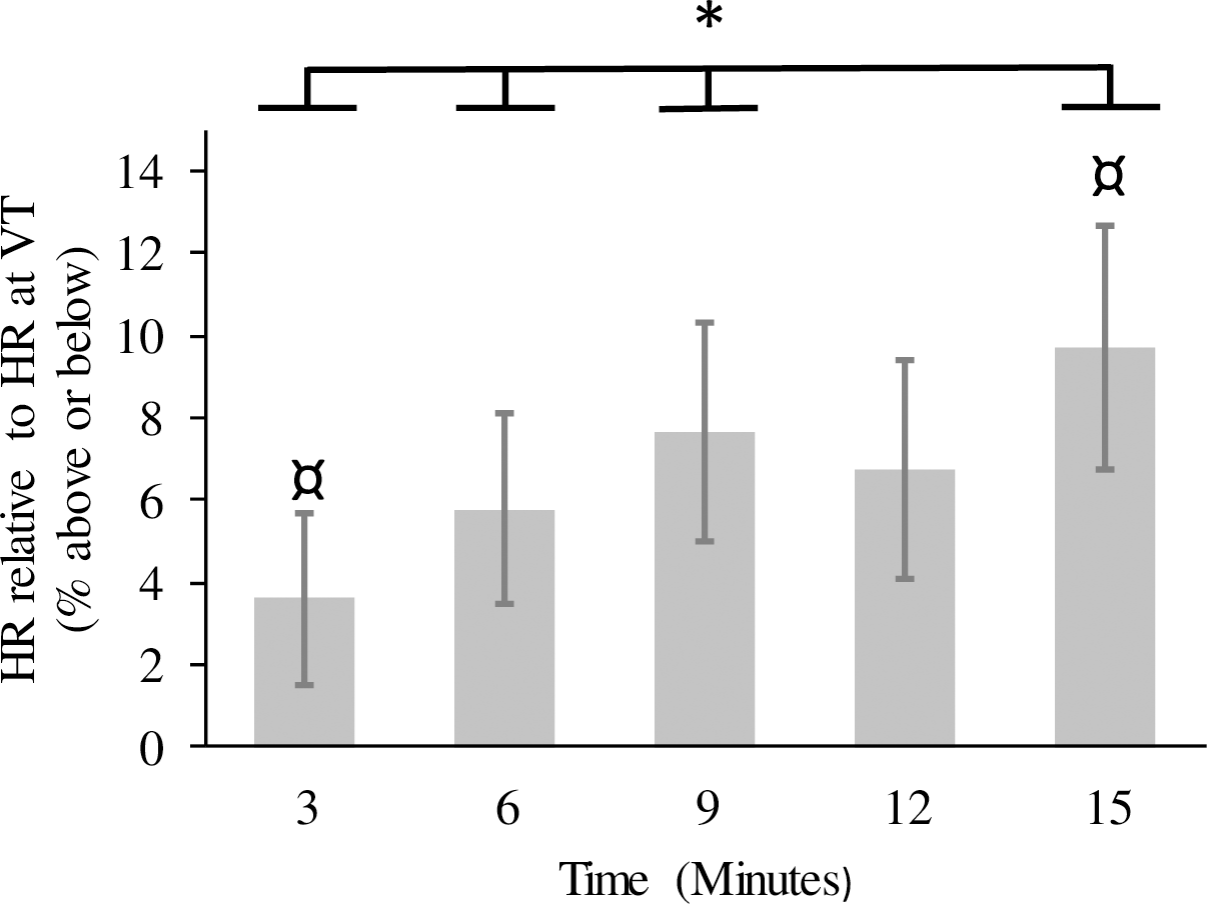
Mean %HR relative to HR at VT for the time main effect. Error bars indicate the standard error of the mean. * significant increase in %HR across time; ¤ different to %HR at 12 min.

**Heart rate.** A significant main effect was found for time, (*F* (2.06, 34.99) = 7.74, *P* < 0.01, η^2^ = 0.13). Pairwise comparisons indicated significant increases across time with the exception of between minutes 9 and 12 (Fig 1). The mode by time interaction was not significant, but the mode main effect approached significance (*F* (2, 34) = 2.87, *P* = 0.07, η^2^ = 0.14) with a medium effect size recorded and HR higher in the track mode relative to game and control modes. Participants on average worked above VT in all three modes (Table 2).

**Fig 2:**
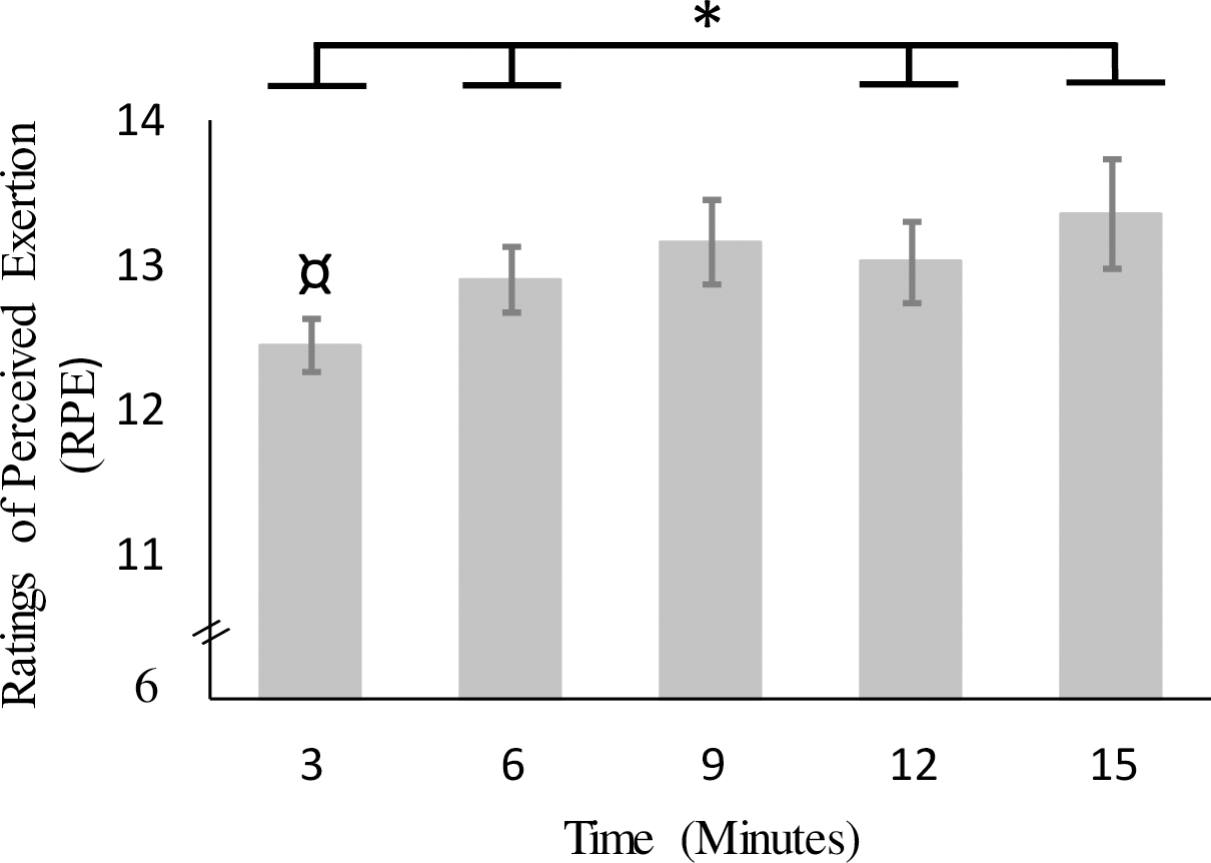
Mean RPE for the time main effect. Error bars indicate the standard error of the mean. * significant increase in RPE across time; ¤ different to RPE at 9 min.

**Rating of perceived exertion.** There were mode *(F* (2, 38) = 3.81, *P* < 0.05, η^2^ = 0.09) and time (*F* (2.44, 46.31) = 6.21, *P* < 0.01, η^2^ = 0.05) main effects. RPE was lower in the control mode than the track and game modes, with no difference between track and game modes (Table 2). RPE increased from minute 3 (*M* = 12.43 ± 1.17) to minute 6 (*M* = 12.83 ± 1.32) and then from minute 12 (*M* = 12.95 ± 1.64) to minute 15 (*M* = 13.28 ± 2.07). The mode by time interaction was not statistically significant (Fig 2).

**Affect.** There was a mode main effect (*F* (2, 38) = 5.4, *P* < 0.01, η^2^ = 0.14) with affect more positive in game mode than track mode, and track more positive than control mode (Table 2). The time main effect and interaction were not statistically significant.

**Dissociation.** The one factor ANOVA resulted in a main effect for mode (*F* (2, 38) = 9.71, *P* < 0.01, η^*2*^ = 0.34) with levels of dissociation higher in game than track mode, which was higher than control mode (Table 2).

**Enjoyment.** Analysis revealed a significant main effect for mode (*F* (2, 38) = 19.67, *P* < 0.01, η^*2*^ = 0.51) with enjoyment significant higher in game than track mode, which was higher than control mode (Table 2).

## Discussion

The purpose of this study was to compare physiological and psychological responses to self-selected exercise intensity on an exergaming ergometer in a control mode (no visual distraction) to a track (woodland circuit with computer generated riders) and game (chasing dragons for treasure) modes. Dependent variables were work rate, HR, perceived exertion, affect, dissociation, and enjoyment. Hypotheses were generally supported, with participants exercising at a higher intensity in the exergaming modes than the control mode, with more positive affect throughout the exercise bouts. Furthermore, dissociation and enjoyment were higher during the exergaming modes and highest during the game mode in support of the hypothesis that the more immersive the mode the more distracting and enjoyable the experience.

Previous research has demonstrated that during self-selected exercise individuals typically select an intensity close to VT [5]. This is an intensity that will lead to physiological gain and a training effect [16]. The present study supports this previous research with participants working on average above their VT regardless of the mode. The mode main effects for work rate and RPE show that participants worked significantly harder in the game and track modes compared to the control mode. Further, in support of previous findings, it appears that participants chose to increase the intensity over the duration of exercise with HR and RPE significantly increasing from 3 minutes to 15 minutes [9, 15, 18]. However, it is of interest to note that there was an absence of a time main effect for work rate. It could be that the variability in work rate selected between participants masked this main effect. Unlike HR (standardised to VT) and RPE, which both take into account the individual’s metabolic state, and are therefore metabolically equivalent between participants, work rate was not standardised. Indeed, absolute work rate varied by over 100 watts at each time point between some participants, which could have masked a work rate time main effect.

As hypothesised, affect remained positive and stable throughout the duration of exercise in all three modes. Further, affective responses were more positive in the game and track modes. This is in line with previous research, which reports that exergaming leads to more positive affective responses than regular exercise [34]. Further, data also support the theory driven research by Jones et al. [22] and Hutchinson et al. [23], which examined the distractive influence of music and video during exercise. In their research, affect was highest with music and video even at intensities 5% and 10% (respectively) above VT compared to no-distraction controls [22, 23]. In the current study, participants chose to exercise between 6% and 9.5% above VT in the game and track modes, with the affective data (positive and stable for mode main effect) supporting the theorised distraction of attention away from the physiological cues of exercise [5].

Previous studies have suggested that the increased work rate during exergaming relative to standard ergometry is due to the distracting nature of exergaming, but they have not always assessed distraction [33]. In the current study, the distracting nature of the exergaming modes is corroborated by the dissociation data which was significantly higher during exergaming. Again, these data are consistent with Jones et al.’s [22] study, which demonstrated that dissociation levels were higher in the music and video condition when compared with the standard condition. The large effect (η^2^ = .34) for mode, with higher levels of dissociation in game mode compared to track mode, also provides support for Warburton et al.’s [32] suggestion that interactive video gaming creates an immersive atmosphere that distracts the participant. Both the track and game mode provide different aspects of immersion. Although, the track mode in this study may be considered a less immersive environment compared to game mode due to its simplistic nature, it does contain potential motivational elements. Participants could choose to race after (and overtake) computer generated riders, if they so wished, or just ride along-side them. However, the cycle pathway was restricted (they could not turn off it, or turn around) whereas in game mode, participants could choose which way they went and which dragon to chase.

The enjoyment data further supports the utility of exergaming and allowing individuals to self-select their exercise intensity. Enjoyment significantly differed between modes, with the largest difference between control mode (*M* = 73.5, *±* 21.6) and game mode (*M* = 104.3, *±* 9.0). Previous research supports that the exercise condition can influence exercise enjoyment, with Kendzierski & DeCarlo [39] reporting higher levels of enjoyment when riding and listening to music, rather than riding and not listening to music. Recent research indicates that enjoyment of physical activity is a stronger predictor of long term activity engagement and efficacy to engage in future activity than actual self-efficacy for physical activity [50]. Consequently, any exercise manipulation (mode or method of regulation) that supports enjoyment has the potential to influence future exercise behaviour [50]. While the exergaming modes led to the highest levels of enjoyment, it is noted that the self-selected exercise on the control mode was on average still experienced as ‘enjoyable’. This is in contrast to research that has compared exergaming and standard ergometry (control mode) when the exercise intensity is prescribed. In Monedero et al. [33], where the exercise intensity was prescribed at 55% peak power output, participants in the control mode reported the exercise to be on average ‘unenjoyable’.

Self-selection of exercise intensity is more ecologically valid (represents what typically happens in a gym environment) and has repeatedly been advocated to support a pleasant affective response to exercise [51] due to the autonomy that it affords the individual to exercise at an intensity that can be achieved [5]. Indeed, when researchers have examined the effect of allowing individuals the autonomy to self-select the intensity, and then removing this autonomy (but keeping the intensity and duration the same), affective responses differ [7]. Although exercising at the same physiological intensity in counterbalanced conditions (self-selected and prescribed), participants reported more positive affective responses during the self-selected condition [7]. It is interesting to note that the present study produced more positive affective responses (*M* = 2.5 ± 0.6; *M* = 2.1 ± 0.6; and *M* = 1.8 ± 0.6) when self-selecting the intensity in game, track and control modes respectively, compared to the prescribed 5% above VT conditions in Jones et al. [22], where affective responses were *M* = 1.9 ± 1.6, *M* = 1.6 ± 1.8, *M* = 0.6, ± 1.7, and *M* = 0.2 ± 2.1, for the music, music and video, video, and control conditions, respectively. So, while affect generally remained positive with the distractions of music or music and video in Jones et al.’s study, the variability in affective response (based on the standard deviations) was considerably greater than that recorded during self-selected exergaming exercise in the current study. In comparison, although the intensities chosen were on average above VT in the current study, they were highly variable (6.2 ± 12%, 9.5 ± 12% and 4.4 ± 14% above HR at VT for the game, track, and control modes respectively). These data highlight that a more positive affective experience, with a lower variability in affective response can be achieved by allowing the individual to self-select the exercise intensity. In line with the Dual-Mode Model [8], the addition of the exergaming modes appears to distract attention from the physiological cues and result in a higher exercise intensity with a maintenance of affect and enhanced enjoyment. However, there is high variability in the degree of distraction, as indicated by the high standard deviations for dissociation (Table 2).

The present study was limited by each exercise bout only lasting 15 minutes, and the inclusion of all three bouts in one experimental session. Whilst 15 minutes is over the 10-minute guideline of continuous activity for cardiovascular gains [4, 48] and modes were randomized across participants (to control for potential order, and fatigue effects), a stronger design would have been for three separate experimental sessions. This would have enabled longer exercise bouts, and ensured that the variability in self-selected intensities were not due to fatigue. We do not know if participants would respond in the same way, physiologically and psychologically, if the exercise bouts were longer (30–40 mins) in duration. Further, as participants chose to exercise on average above VT, and at higher intensities in the exergaming modes, caution may need to be taken in adopting these modes of exercise and method of regulation for individuals with health conditions that contraindicate exercise above a moderate intensity.

Notwithstanding these limitations, the study confirmed that when healthy individuals were allowed to self-select their exercise intensity, they selected an intensity close to (and on average above) their VT and during exercise they reported a positive affective response. When the individuals were allowed to self-select their intensity during exergaming (game and track modes), they worked harder, reported more positive affect and greater dissociation. They also reported more exercise enjoyment. Together these data support the utility of exergaming from a behaviour change perspective and for physiological and psychological health, with research warranted in the application of exergaming in exercise interventions.
